# Early and Unplanned Readmission of Patients After Thoracic Surgery Operations

**DOI:** 10.7759/cureus.71190

**Published:** 2024-10-10

**Authors:** Hassan Edward Bakali, Mehmet Sezer, Saltuk Burhan Dal, Ufuk Cagirici, Tevfik Ilker Akcam, Ali Ozdil, Ayse Gul Ergonul, Kutsal Turhan, Alpaslan Cakan

**Affiliations:** 1 Thoracic Surgery, Ege University Faculty of Medicine, Izmir, TUR; 2 Thoracic Surgery, Acibadem - Izmir Kent Hospital, Izmir, TUR

**Keywords:** minimally invasive surgery, postoperative complications, pulmonary resection, readmission, thoracic surgery

## Abstract

Background

Thoracic surgery patients face unique postoperative challenges, particularly those undergoing lung resections. This study aimed to identify common complications and their management within the 30-day postoperative period, as well as investigate the relationship between early postoperative complications and unplanned outpatient follow-ups.

Methods

A retrospective analysis was conducted on 947 patients who underwent thoracic surgery between January 2020 and December 2023. Of these, 168 patients who had unscheduled follow-ups were included in the final analysis.

Results

The most common surgical procedure was pulmonary resection (68.5%), with prolonged air leaks (19.6%) being the most frequent postoperative complication. Unplanned outpatient visits were primarily due to dyspnea (38.1%) and surgical site complications (33.9%). While the rate of readmission did not significantly differ between complication-free and complicated patients (p=0.070), patients with complications were 2.117 times more likely to be readmitted (p=0.044). Surgical technique impacted postoperative outcomes, with minimally invasive surgery associated with fewer complications and shorter hospital stays compared to open thoracotomy.

Conclusion

Our findings emphasize the need for tailored postoperative care and diligent follow-up, especially for patients at higher risk of readmission due to complications such as pneumothorax, empyema, and prolonged air leaks.

## Introduction

Thoracic surgery patients require specialized care during the postoperative period. The type of surgery and the patient’s comorbidities pose significant challenges for postoperative management and complications. This is especially important for patients undergoing lung resections [1,2]. With a range of procedures including lung resections (both anatomic and non-anatomic), mediastinal surgery, pleural surgical procedures such as decortication for mesothelioma and empyema, pleural effusion drainage, pleural biopsies, and chest wall resections and reconstructions, postoperative care needs to be tailored for each patient based on their type of procedure and risk factors for postoperative complications.

While the risk factors for postoperative complications in thoracic surgery patients are well-known, few studies have explored the frequency of complications within the 30-day postoperative period [1,3]. Discussing these complications and their management may also provide insights into how to evaluate and manage these patients in the preoperative period. They might also serve as a real-world guide to postoperative care and management.

This study aimed to explore the common complaints of patients, complications, and management of these complications within the 30-day postoperative period in patients who have undergone different procedures in our thoracic surgery clinic. We also investigated whether there is a relationship between early postoperative complications and unplanned outpatient clinic follow-ups by patients.

## Materials and methods

A retrospective study was conducted to identify common late postoperative complications in thoracic surgery patients. The study examined their main complaints on follow-up, the place of admission (i.e., outpatient department [OPD] or emergency department [ED]), the type of intervention when needed, and whether rehospitalization or reoperation was required.

A database search of all the patients who had been admitted to our thoracic surgery clinic between January 2020 and December 2023 was conducted. Patients who underwent different surgical procedures during this period were identified and included in the study. The patients’ demographic features, comorbidities, smoking history, type and method of operation (open or minimally invasive), postoperative complications, and hospital length of stay (LOS) were noted. The patients’ chest X-rays on the day of the follow-up were also evaluated, and any remarkable radiological findings were noted.

Patient data were accessed and collected from our hospital’s electronic patient information management system and a personal health record system used in Turkey (E-Nabız), implemented by the Ministry of Health of the Republic of Turkey. It compiles each patient’s data from most of the private and government hospitals in Turkey under one roof for easy access by both the patients and physicians.

Patients who underwent different thoracic surgical procedures at our thoracic surgery clinic between January 2020 and December 2023 were included in the study. Patients who underwent surgery for trauma or whose hospital records were inaccessible, patients with early mortality, i.e., less than 30 days, patients who underwent procedures under local anesthesia or lung transplants, and patients in the pediatric age group at the time of surgery were excluded from the study.

A total of 947 patients who met the inclusion criteria were included in the study. The patients’ hospital records were accessed, and those who had an uneventful 30-day postoperative follow-up were picked out and put aside. Those who had complaints on their follow-up or had unscheduled follow-ups either at our OPD or the ED were noted. Postoperative patients in our clinic have two scheduled follow-ups: on day 10 after discharge and on the 30th postoperative day. Therefore, any follow-up outside the above-mentioned routine was accepted as unscheduled. A total of 168 patients meeting this criterion were then included in the final study. Their records were further explored to identify their complaints on the day of the follow-up, radiological findings, interventions, or rehospitalizations, if any.

Data were analyzed using SPSS Version 23 (IBM Corp., Armonk, NY). The Shapiro-Wilk test and the Kolmogorov-Smirnov test were used to test adherence to a normal distribution. The chi-squared test and Yates correction were used to compare categorical variables between groups. The Mann-Whitney U test was used to compare unevenly distributed data by binary groups. The effect of independent variables on rehospitalization was analyzed usinga binary logistic regression analysis. Analysis results were presented as mean ± standard deviation (SD) and median (minimum-maximum) for quantitative data and frequency (percentage) for categorical data. The significance level was set at p<0.05.

## Results

A total of 168 postoperative patients were analyzed in this study. Of these, 111 (66.1%) were men, and the mean age was 57.56 (SD: ±13.85) years. Most patients (50.6%) were aged 60 years or older. In total, 60.7% of patients had comorbidities, the most common of which were cardiovascular (37.5%) and endocrinologic, with the others making up the remaining 21.4%. The mean postoperative hospital LOS was 7.56 days (Table 1).

**Table 1:** Sociodemographic and intervention-related characteristics *Multiple responses MIS, minimally invasive surgery; LOS, length of stay; OPD, outpatient department; ED, Emergency Department

	Frequency (n) / mean ± SD	Percentage (%) / median (min. - max.)
Sociodemographic/clinical characteristics		
Sex		
Male	111	66.1
Female	57	33,9
Age (years)	57.56±13.85	60.00 (19.00–86.00)
Age group		
<60	83	49.4
60 and up	85	50.6
Comorbidity		
Absent	66	39.3
Present	102	60.7
Comorbidities*		
None	66	39.3
Cardiovascular	63	37.5
Endocrinologic	36	21.4
Cerebrovascular	3	1.8
Pulmonary	18	10.7
Miscellaneous	36	21.4
Prior malignancy		
Absent	118	70.2
Present	50	29.8
Smoking history		
None	111	66,1
Active	57	33.9
Procedure type*		
Lung resection	115	68.5
Pleural surgical procedure	20	11.9
Mediastinal surgery	13	7.7
Chest wall surgery	11	6.5
Miscellaneous	11	6.5
Surgical technique		
Open thoracotomy	80	47.6
MIS	88	52.4
Postoperative hospital LOS (days)	57.56±13.85	60.00 (19.00–86.00)
Postoperative complications		
Absent	96	57.1
Present	72	42.9
Place of admission		
OPD	146	86.9
ED	22	13.1
Intervention*		
Pleural drainage catheter	27	16.1
Observation	26	15.5
Antibiotics/analgesics	98	58.3
Wound debridement and suturing	35	20.8
Seroma/hematoma drainage	4	2.4
Pleural lavage	5	3
Miscellaneous	20	11.9
Readmission		
Yes	43	25.6
No	125	74.4

The most common surgical procedure was pulmonary resection (68.5%), of which 52.4% were performed using minimally invasive techniques. The rate of postoperative complications was 42.9%, with prolonged air leaks (PALs) as the most common complication encountered. The unplanned outpatient admission rate was 86.9%, while the ED admission rate was 13.1%. The rate of readmission was 25.6% with 43 patients (Table 2). The most common complaints on presentation were dyspnea (38.1%) and discharge/opening/swelling at the surgical site (33.9 %) (Table 2).

**Table 2:** Complications, complaints, and radiologic abnormalities of the patients *Multiple responses RF; renal function

Clinical characteristics	Frequency (n)	Percentage (%)
Complications*		
None	96	57.1
Prolonged air leak	33	19.6
Pneumothorax	4	2.4
Empyema	5	3
Blood transfusion	12	7.1
Cardiac	12	7.1
RF/electrolyte imbalance	3	1.8
Pneumonia/atelectasis	10	6
Miscellaneous	17	10.1
Hemorrhage/reoperation	5	3
Complaints on admission*		
Cough/expectoration	24	14.3
Dyspnea	64	38.1
Wound site swelling/opening/discharge	57	33.9
Chest/incision site pain	24	14.3
Malaise/fever	15	8.9
Hoarseness	5	3
Miscellaneous	10	6
Radiologic abnormality*		
None	66	39.3
Pleural effusion	56	33.3
Pneumonia/atelectasis	24	14.3
Pneumothorax	25	14.9
Subcutaneous emphysema	7	4.2
Miscellaneous	7	4.2

There was a difference in the distribution of postoperative complications according to the surgical method (p=0.036). Complications were observed in 51.3% (41 patients) of the open thoracotomy group and 35.2% (31 patients) of the minimally invasive surgery (MIS) group. The observed complications differed by surgical approach (p=0.001). The open thoracotomy approach had a rate of 11.3%, whereas the MIS approach had a rate of 3.4% of cardiac complications (Table 3). There was a difference in the median length of postoperative hospital stay depending on the surgical technique (p=0.005). The median length of stay for open thoracotomy was 5.00, whereas the median length of stay for MIS was 4.00 (Table 4).

**Table 3:** Comparison of complications according to surgical technique *Chi-square test. **Multiple responses. ^a,b^No difference was found between methods with the same notation on multiple comparisons. PAL, prolonged air leak; RF; renal function

	Surgical technique, frequency (percentage)		p-Value*
	Open thoracotomy	MIS	
Postoperative complications			
None	39 (48.8)	57 (64.8)	0.036
Present	41 (51.3)	31 (35.2)	
Complications**			
None	39 (48.8)^a^	57 (64.8)^b^	0.001
PAL	15 (18.8)	18 (20.5)	
Pneumothorax	1 (1.3)	3 (3.4)	
Empyema	3 (3.8)	2 (2.3)	
Blood transfusion	12 (15)	0 (0)	
Cardiac	9 (11.3)^a^	3 (3.4)^b^	
RF/electrolyte imbalance	3 (3.8)	0 (0)	
Pneumonia/atelectasis	6 (7.5)	4 (4.5)	
Miscellaneous	10 (12.5)	7 (8)	
Hemorrhage/reoperation	2 (2.5)	3 (3.4)	

**Table 4:** Comparison of postoperative hospital length of stay according to surgical technique *Mann-Whitney U test MIS, minimally invasive surgery

Surgical technique	Mean ± SD	Median (min.–max.)	p-Value
Open thoracotomy	7.00±7.91	5.00 (1.00–63.00)	0.005*
MIS	4.90±3.96	4.00 (1.00–26.00)	

Though there was no statistically significant difference in the rate of readmission according to postoperative complications (p=0.070), readmission was observed in 19.8% of the complication-free patients and in 33.3% of those with complications. There was a difference in the rate of readmission by complication (p=0.002). Readmission was observed in 19.8% of the patients without complications, 42.8% of those with PAL, 75% of those with pneumothorax, 60% of those with empyema, 8.3% of those with postoperative blood transfusion history, 50% of those with cardiac complications, 10% of those with pneumonia/atelectasis, and 40% of those with postoperative hemorrhage/reoperation history. There were no differences in readmission rates based on surgical technique, comorbidity, or additional malignancies (p>0.050) (Table 5).

**Table 5:** Comparison of readmission rates according to variables *Chi-square test. **Yates correction. ***Multiple responses, frequency (percentage) MIS, minimally invasive surgery; PAL, prolonged air leak; RF, renal function

	Readmission		p-Value
	No	Yes	
Postoperative complication			
None	77 (80.2)	19 (19.8)	0.070**
Present	48 (66.7)	24 (33.3)	
Surgical technique			
Open thoracotomy	60 (75)	20 (25)	1.000**
MIS	65 (73.9)	23 (26.1)	
Comorbidity			
None	48 (72.7)	18 (27.3)	0.826**
Present	77 (75.5)	25 (24.5)	
Prior malignancy			
None	84 (71.2)	34 (28.8)	0.202**
Present	41 (82)	9 (18)	
Comorbidities***			
None	48 (72.7)	18 (27.3)	0.167*
Cardiovascular	48 (76.2)	15 (23.8)	
Endocrinologic	31 (86.1)	5 (13.9)	
Cerebrovascular	1 (33.3)	2 (66.7)	
Pulmonary	11 (61.1)	7 (38.9)	
Miscellaneous	29 (80.6)	7 (19.4)	
Complications***			
None	77 (80.2)	19 (19.8)	0.002*
PAL	19 (57.6)	14 (42.4)	
Pneumothorax	1 (25)	3 (75)	
Empyema	2 (40)	3 (60)	
Blood transfusion	11 (91.7)	1 (8.3)	
Cardiac	6 (50)	6 (50)	
RF/electrolyte imbalance	3 (100)	0 (0)	
Pneumonia/atelectasis	9 (90)	1 (10)	
Miscellaneous	13 (76.5)	4 (23.5)	
Hemorrhage/reoperation	3 (60)	2 (40)	
Procedure type***			
Lung resection	84 (73)	31 (27)	0.814*
Pleura surgical procedure	17 (85)	3 (15)	
Mediastinal surgery	9 (69.2)	4 (30.8)	
Chest wall surgery	9 (81.8)	2 (18.2)	
Miscellaneous	8 (72.7)	3 (27.3)	

There was also no difference in the median age by readmission. The median age was 59 for those without readmissions and 61 for those with readmissions (Table 6). The effect of independent variables on readmission was analyzed by binary logistic regression analysis with univariate and multiple models. In the univariate model, the risk of readmission was 2.026 times higher in those with postoperative complications than in those without postoperative complications (p=0.048). In the multiple models, the risk of readmission was 2.117 times higher in those with postoperative complications compared to non-complicated patients (p=0.044). The other variables did not have an effect on readmission (p>0.050) (Table 7).

**Table 6:** Comparison of age by readmission *Mann-Whitney U test

Readmission	Mean ± SD	Median (min.–max.)	p-Value
No	57.90±13.53	59.00 (20.00–86.00)	0.752*
Yes	56.56±14.88	61.00 (19.00–80.00)	

**Table 7:** Analysis of the effect of the independent variables on readmission

	Univariate		Multiple	
	OR (%95 CI)	p-Value	OR (%95 CI)	p-Value
Age	0.993 (0.969–1.018)	0.582	0.991 (0.964–1.019)	0.527
Comorbidity (reference: none)	0.866 (0.428–1.752)	0.689	0.81 (0.369–1.779)	0.600
Prior malignancy (reference: none)	0.542 (0.238–1.236)	0.146	0.534 (0.225–1.267)	0.155
Surgical technique (reference: open thoracotomy)	1.062 (0.53–2.126)	0.866	1.293 (0.624–2.68)	0.489
Postoperative complication (reference: none)	2.026 (1.005–4.087)	0.048	2.117 (1.02–4.39)	0.044

## Discussion

The most common complications and their frequency in evaluating postoperative complications in thoracic surgery patients are well known [4]. However, not a lot of studies have been conducted on the most common causes of unplanned outpatient visits or readmissions in thoracic surgery. Bouabdallah et al. [5] in their study evaluating unplanned readmission and survival after video-assisted thoracic surgery (VATS) and open thoracotomy in patients with non-small-cell lung cancer found that the most common complications associated with unplanned readmission at 30 days were pulmonary, 40.6% for VATS and 44.6% for open thoracotomy, followed by cardiac complications. Pleural effusion (33.9% for VATS and 39% for open thoracotomy) and pneumonia (24.7% for VATS and 17.5% for open thoracotomy) were the most common pulmonary complications. Polites et al. [6] in their multi-institutional cohort study of 30-day postoperative readmission rates in pediatric patients who had general and thoracic surgery found the readmission rate of thoracic surgery patients to be 2.7% (64 patients) compared to 2.8% (653 patients) for general surgery patients. Postoperative hospital LOS was found to be the strongest predictor of readmission, with children with a postoperative hospital LOS of >4 days three times more likely to be readmitted than children discharged within two days (p<0.001) for both groups [6]. Uchida et al. [7] explored factors associated with unexpected readmission after lung resection and found that postoperative hospital LOS was signiﬁcantly longer in the readmission group (8.6 ± 10.6 days) than in the no readmission group (5.0 ± 5.1 days; p<0.0001; t-test). They also found refractory air leak as a risk factor readmission (23.2%, vs. 5.8%; p<0.001), similar to other studies [8].

Our study group was much more heterogeneous, consisting of a broad range of thoracic surgical procedures. Lung resections were the most common procedure (68%). The most frequent postoperative complication was PAL (19.6%). This can be attributed to lung resections being the most common procedure performed at the clinic. Shortness of breath (38.1%) was the most common complaint responsible for unplanned visits to the OPD or ED, followed by wound site swelling/dehiscence/discharge (33.9%). In the evaluation of the patients’ chest X-rays, 39.3% of the images were consistent with postoperative changes. Pleural effusion (33.3%), followed by pneumothorax (14.9%) and pneumonia/atelectasis (14.3%) were the most common pathologies discovered on imaging. However, most of the patients needed no intervention, needing only antibiotics/analgesia (58.3%) and follow-up with no medication (15.5%). Shortness of breath and wound dehiscence/discharge were the most common reasons for unplanned visits, and pleural effusion was the most common radiopathology on evaluation, with 16.1% of the patients requiring a pleural drainage catheter insertion and 20.8% of them requiring wound debridement and suturing.

When we look over the factors affecting readmission of the patients, the presence of postoperative complications itself was not a direct factor responsible for readmission, as the difference between complicated and non-complicated patients, 24 (33.3%) and 19 (19.8%) respectively, was not significant (p=0.070). However, the presence of postoperative complications meant that the risk of readmission was 2.026 and 2.117 times higher in the univariate and multivariate models, respectively, compared to postoperative complication-free patients (p=0.048 and p=0.044). When the types of complications and whether they had an effect on readmission were investigated, interesting findings were obtained: three (75%) with pneumothorax, six (50%) with cardiac complications, three (60%) with empyema, and 14 (42.4%) with PAL as postoperative complications required readmission, which was statistically significant (p=0.002). Similarly, Brown et al. [9] in their multicenter study of 39,734 post-lobectomy patients found that pulmonary embolism and empyema, followed by pleural effusion and pneumothorax, were associated with the greatest odds of readmission. Prolonged hospital LOS, comorbidities, and types of surgery had no statistically significant effect on readmission. Assi et al. [10] evaluated readmission in post-lobectomy patients and found that readmission was not affected by surgical technique. We also had similar findings in our cohort study, as the surgical approach had no statistically significant effect on readmission.

The study also showed that, as expected, MIS had fewer postoperative complications compared to open thoracotomy, 35% and 51.3%, respectively (p=0.001), concurrent with the literature [9,10]. Also concurrent with literature findings was the postoperative hospital LOS results, with the median being 4 days for MIS and 5 days for open thoracotomy (p=0.005). These findings are also similar to the studies in literature, if not providing a different perspective [11-14].

Limitations

The study, being a database search, relied on information from our own university hospital and the national system. Lack of information or lack of access led to some patients being excluded from the study. Being a single-center study is also one of its limitations. Larger multi-database studies can paint a much more accurate picture. Our study cohort was diverse, including all general thoracic surgeries rather than focusing on specific surgical groups, such as lobectomies or lung resections, similar to many studies in the literature, though an argument can be made of the uniqueness of the study in this format.

## Conclusions

In our study, we found that the most common complaint responsible for unplanned visits to our thoracic surgery clinic by postoperative patients was shortness of breath and surgical site opening or discharge, with most of the patients requiring administration of antibiotics or analgesia as intervention. Patients who had MIS had statistically, significantly fewer postoperative complications than open thoracotomy patients. Pneumothorax, empyema, cardiac complications, and PAL were associated with a greater rate of readmission. The presence of postoperative complications was associated with a 2.026- and 2.117-fold risk of readmission on univariate and multivariate analysis than complication-free patients. Our results highlight other similar literature findings while providing a different perspective on patient management and follow-up. The importance of preoperative evaluation, perioperative and postoperative care, and follow-up cannot be stressed enough for thoracic surgery patients. Patients identified as having a high risk for readmission require meticulous postoperative management and follow-ups.
